# Impacts of adding sucrose or trehalose to extenders with different glycerol concentrations on freezablility and fertility of buffalo bull semen

**DOI:** 10.1007/s11259-024-10573-z

**Published:** 2024-11-20

**Authors:** Wael A. Khalil, Ragaey M. El-Deghaidy, Abdelaziz M. Sakr, Ayman A. Swelum, Sameh A. Abdelnour, Mostafa A. El-Harairy

**Affiliations:** 1 Department of Animal Production, Faculty of Agriculture, Mansoura University, Mansoura, 35516 Egypt; 2 Animal Production Research Institute, Agriculture Research Centre, Ministry of Agriculture, Dokki, 12619 Giza Egypt; 3 Department of Theriogenology, Faculty of Veterinary Medicine, Zagazig University, Zagazig, 44519 Egypt; 4 Department of Animal Production, Faculty of Agriculture, Zagazig University, Zagazig, 44511 Egypt

**Keywords:** Buffalo semen, Low glycerol, Sperm quality, Cryopreservation

## Abstract

This experiment was conducted to determine the most suitable glycerol concentration (3 or 6%) and/or non-penetrating cryoprotectants (trehalose and sucrose) for the cryopreservation of buffalo semen, with the aim of enhancing the cryopreservation protocol. Semen of Egyptian buffalo were pooled and diluted with eight Tris extenders supplemented with either 6% glycerol (control group, GL6), 3% (low level, GL3), sucrose (SU, 50 mM), trehalose (TR, 50 mM), 6% glycerol together with 50 mM of sucrose (GL6SU) or 50 mM of trehalose (GL6TR), and 3% of glycerol together with 50 mM of sucrose (GL3SU) or 50 mM of trehalose (GL3TR), then frozen following the standard protocol. Findings indicated that GL3 extender resulted in the highest values of progressive motility, sperm kinematics, sperm membrane integrity, and viability of post-thawed semen (37 °C for 30 s). On the contrary, the Tris extender enriched only with SU and TR groups had the lowest values of sperm quality compared to the other groups (*p* < 0.05). All GL supplemented groups showed higher intact acrosome levels and lower detached acrosome and dead sperm with intact acrosome compared to those with TR and SU alone (*p* < 0.05). A significant increase in viable sperm was observed in the GL3, GL6, and GL3SU groups compared to the other groups (*p* < 0.05). The Tris extender supplemented with low glycerol (3%) significantly reduced the levels of MDA. In the in vivo fertility trial, it was shown that the pregnancy rate was higher in the GL6SU group (72%) than in the GL3SU group (68%; *p* > 0.05). Collectively, these results suggest that there is potential in using low glycerol (3%) as a cryoprotective agent in the medium for buffalo sperm cryopreservation without significant adverse effects compared to the addition of 6% glycerol. This study supported the sustainability of materials used in assisted reproductive technology by reducing the glycerol content in the freezing medium. Further research is needed to confirm this hypothesis.

## Introduction

Buffaloes play an important role in the livestock industry, contributing significantly to the production of milk, as well as providing meat and draught power (Baruselli et al. 2023). Cryopreservation of buffalo semen has been instrumental in enhancing the genetic quality of this species through artificial insemination techniques (Khalil et al. 2023). Sperm cryopreservation is a valuable method to facilitate breeding management in livestock, propagate superior genotypes, and maximize production efficiency (Sharafi et al. 2022). Thereafter, embryologists have been working vigorously to improve the cryopreservation process of buffalo semen. There are many hurdles related to sperm cryopreservation including the negative impact of the cryoprotectants and the increased levels of oxidative stress, which can affect the sperm fertilizing capacity (Abdelnour et al. 2024; Ugur et al. 2019). Semen cryoprotectants are chemical substances that protect cells against cryo-injury during cryopreservation. Among them, glycerol (GL) is a penetrating cryoprotectant commonly used in various species to provide intracellular protection for sperm cells (Lin et al. 2023; Swelum et al. 2011). Nonetheless, it can have cytotoxic impacts on sperm functions, impacting their biological characteristics and fertilization capacity. This has been observed in humans (Gao et al. 1993), hens (Lin et al. 2023), horses, donkeys (Macías García et al. 2012), and pigs (Gutiérrez-Pérez et al. 2009).

In buffalo, a 6% concentration of glycerol (GL) is the standard protocol that used for sperm cryopreservation (Shah and Andrabi 2021). However, this concentration has been shown to have detrimental effects on sperm health and functionality in buffaloes (Akhter et al. 2020; Kadirvel et al. 2012), resulting in a significant reduction in fertility. The toxicity resulting from the use of a high concentration of GL during buffalo sperm cryopreservation may be attributed to osmotic stress. This emphasizes the need to develop improved sperm cryopreservation protocols with a lower glycerol concentration. Research has shown that using a lower concentration of GL (1.75%) in conjunction with other penetrating cryoprotectants like DMSO (1.75%) can improve the effectiveness and in vivo fertility of cryopreserved buffalo sperm (Shah et al. 2016).

On the other hand, disaccharide sugars such as sucrose (SU) and trehalose (TR) are extracellular cryoprotectants that do not penetrate the cell membrane. The addition of TR (100 mM) and SU (60 mM) along with vitamin E (2 mM) to Tris-egg yolk extender significantly improved the quality of frozen-thawed ram sperm (Rostami et al. 2020). In humans, TR (50 mM) has been shown to enhance post-thaw sperm quality (Suksai and Dhanaworavibul 2019). Additionally, the use of SU has demonstrated a significant impact on cryopreserved chicken sperm and in vivo fertility (Thananurak et al. 2019). In light of conflicting findings from previous studies regarding the optimal levels of glycerol (GL), we hypothesized that decreasing glycerol concentration and incorporating SU or TR as extracellular cryoprotectants would maintain or improve post-thaw sperm quality, antioxidant status, and fertility outcomes. Despite extensive research on this topic, the potential cryoprotective effects of low levels of GL, either alone or in combination with SU or TR, have not been explored in the context of freezing buffalo sperm. This study aims to comprehensively compare the effects of high or low concentrations of GL in semen extenders, either alone or in combination with SU or TR as cryoprotectants, on sperm characteristics, kinematic parameters, apoptosis-like changes, acrosome reaction, antioxidant and oxidative biomarkers, and in vivo fertility (pregnancy rate) of cryopreserved buffalo bull sperm.

## Materials and methods

### Ethical approval

All animal and trial and procedures were reviewed and approved by the Animal Ethics Committee of Zagazig University, based on the rules of the Institutional Animal Use and Care Committee (IACUC) with Approval Number: ZU IACUC/2/F/173/2022. Furthermore, in accordance with the ARRIVE guidelines, the use and care of animals have adhered to these rules.

### Animal, semen extender formulation, and experimental design

The proven fertility Egyptian buffalo bulls (*n* = 5, aged 3–5 years) from Mahalet Mussa Station, Kafr El-Sheikh Governorate, belonging to the Ministry of Agriculture, Animal Production Research Institute, Agriculture Research Center, Egypt, were used in the existing experiment.

All animals were housed under optimal conditions for feeding, welfare, and management. The extender consisted of 3.028 g of Tris, 1.675 g of citric acid, and 1.25 g of fructose in 100 mL distilled water. Moreover, penicillin 100 IU/mL, and streptomycin 100 µg/mL, and 20% egg yolk (v/v) were added (Tris-based extender). Based on the concentration of glycerol (GL) (3% or 6%) and the addition of either 50 mM sucrose (SU) or trehalose (TR), a total of eight extenders were formed as follows:

1. The GL6 group: the Tris-based extender was supplemented with 6% glycerol.
2. The GL6SU group: the Tris-based extender was supplemented with 6% glycerol + 50 mM sucrose.
3. The GL6TR group: the Tris-based extender was supplemented with 6% glycerol + 50 mM trehalose.
4. The GL3 group: the Tris-based extender was supplemented with 3% glycerol.
5. The GL3SU group: the Tris-based extender was supplemented with 3% glycerol + 50 mM sucrose.
6. The GL3TR group: the Tris-based extender was supplemented with 3% glycerol + 50 mM trehalose.
7. The SU group: the Tris-based extender was supplemented with 50 mM sucrose.
8. The TR group: the Tris-based extender was supplemented with 50 mM trehalose.

### Semen collection and processing

Following the guidelines and standard procedures of the ILMTC (International Livestock Management Training Center), Sakha, Kafr El-Sheikh Governorate, Egypt, semen ejaculates were collected from five healthy buffalo bulls (aged 3–5 years) using an artificial vagina in a routine system at this institution. A total of 30 ejaculates were selected and accepted for this experiment based on the following criteria: ≥70% sperm motility, ≤ 15% abnormalities, and ≥ 10^9^ sperm/mL. Samples with minor values of the previous criteria were discarded. The accepted ejaculates were pooled to minimize the individual difference and diluted based on initial sperm concentration. Each pool aliquot was diluted by one of the eight experimental extenders to a final concentration of 100 × 10^6^ sperm/mL. The diluted samples were gradually cooled from 37 °C to 5 °C within 2 h and equilibrated for 4 h before packing into straws (0.25 mL, IVM technologies, France). The straws were placed 4 cm above the surface of liquid nitrogen for 10 min and then stored in liquid nitrogen. Semen was cryopreserved for at least one month before post-thawing assessments.

- Semen evaluation

Semen samples were assessed under different conditions, including after being equilibration at 5 °C for 4 h (just before freezing), after being thawed at 37 ˚C for 30s, and post-thawing and incubation at 37 °C and 5% CO_2_ for 2 h.

### Sperm motility and kinematic parameters

The sperm progressive motility (PM) was evaluated by a phase-contrast microscope (DM 500, Leica, Switzerland) with a hot stage adjusted to 37 °C. In brief, 10 µL aliquots (have one million sperm) of diluted samples were located on pre-heated slides and covered with a cover slip. The values of PM were noticed in various fields (4–5 fields) of the microscope for each semen sample at 100x magnification. We assessed 6 samples in each treatment.

To assess sperm kinematic feature by Computer Assisted Sperm Analysis (CASA), the procedure described by Dessouki et al. (2022) was followed. The software CASA sperm analyzer (Sperm Vision; Ref: 12520/5000; Minitube Hauptstrae 41. 84184 Tiefenbach, Germany) was used to give more data on the live pictures of several sperm motion items portrayed by the Olympus computer-assisted microscope (Hamburg, Germany). Moreover, the system was set up at 37 °C. The microscope was connected with a rapid scan digital camera to view (60 frames per second) and denote images at 60 Hz under ×4 dark-field illumination. In each group, around 1500 spermatozoa were investigated utilizing CASA. The following motion parameters were recorded: distance average path (DAP, µm), distance curved line (DCL, µm), distance straight line (DSL, µm), velocity average path (VAP, µm/sec), velocity curved line (VCL, µm/sec), velocity straight line (VSL, µm/sec), straightness (STR, % = VSL/VAP), linearity (LIN, % = VSL/VCL), wobble (WOB, % = VAP/VCL), amplitude of lateral head displacement (ALH, µm), and beat cross frequency (BCF, Hz).

### Viability and abnormalities

Morphological sperm cell abnormalities and sperm viability were assessed using eosin-nigrosine staining (Moskovtsev and Librach 2013). In brief, 10 µL of extended semen samples were mixed with 10 µL of pre-warmed eosin 5%-nigrosin 10%, stain for one minute and then immediately smeared on a glass slide. Using a light microscope (Leica DM 500, Schweiz, X400) we evaluated at least two hundred sperm in each sample. Sperm cells that were not stained with eosin (i.e. remained greyish white) were categorized as living cells, while those stained with eosin (i.e. with red head) were identified as dead spermatozoa. Furthermore, we recorded the percentages of sperm cells with abnormal tail and head morphology as described by Khan and Ijaz (2007).

### Sperm membrane integrity

Sperm membrane integrity was assessed using the Hypo-Osmotic Swelling Test (HOST) (Khalil et al. 2019). Briefly, 50 µL of extended semen samples and 500 µL of HOST solution (6.75 g/L fructose and 3.67 g/L sodium citrate with 75 mOsmol/L) were mixed and incubated for 30 min at 37 °C in a water bath. After that, 10 µL of the mixture was placed on a microscope slide and covered with a coverslip and observed under a phase-contrast microscope (DM 500; Leica, Switzerland) at a 400× magnification. According to the procedure of Correa and Zavos (1994), sperm were assessed for their ability to swell in HOST, and sperm with coiled or swollen tails were reported to possess an intact plasma membrane. A total of 200 sperm cells were estimated for their swelling capability in HOST.

### Evaluation of acrosome reaction

Extended semen samples (200 µL aliquots) were mixed with equal volume of 0.2% trypan blue and incubated at 37 °C for 10 min (Kant et al. 2017). After incubation, sperm were diluted with albumin-free modified Brackett and Oliphant medium (2 mL) and centrifugated at 700 g for 5 min. The supernatant was removed, and the pelleted sperm was resuspended in 2–3 mL of the same media and centrifuged again 2–3 times until the suspension was clear or light blue. Next, the sperm suspension (approximately 20µL) was positioned on a slide and smeared with another glass slide. The slides were quickly dried on a warming platform (at 40 °C). The sperm was stained with a 10% Giemsa solution for 35–40 min and washed with distilled water and left to air dry. To determine viability and acrosome reaction, the slides were examined under brightfield microscopic (1000x), and 100 spermatozoa were randomly selected per slide. The following criteria were used for classification.

- Live sperm with intact acrosome: post acrosome is white, and acrosome is bright pink /purple.
- Live sperm with detached acrosome: post acrosome is white, and acrosome is white (true acrosome reaction).
- Dead sperm with intact acrosome: post acrosome is blue or dark blue and acrosome is dark pink /purple.
- Dead sperm with detached acrosome: post acrosome is blue, and acrosome is white/gray, white (false acrosome reaction).

### Sperm apoptosis assessment

In this experiment, Annexin V staining was used to assess sperm apoptosis (Hoogendijk et al. 2009). Extended semen (1 mL) was suspended in binding buffer (2 mL) and mixed well. The suspension (100 µL) was then blended with Propidium Iodide (5 µL; PI) and Annexin V (5 µL; fluorescein isothiocyanate label), and incubated in a dark place at 37 °C for at least 15 min. After incubation, the spermatozoa cells were suspended in a binding buffer (200 µL). Flow cytometry analysis was used to acquire and examine the records using software (Becton Dickinson) and an Accuri C6 Cytometer (Biosciences, San Jose, USA).

The proportions of positive or negative Annexin V (A-/A+), PI (PI-/PI+), and double-positive cells were evaluated. Moreover, corresponding to Khan et al. (2009) analysis, the sperm were sorted into four classes as follows: viable cells: membrane dysfunction and no fluorescence signal (A-/PI-); sperm cells with early apoptosis: viable cells categorized with Annexin V but without PI (A+/PI-); sperm cells with apoptosis: dead cells categorized with Annexin V and PI and with damaged permeable membranes (A+/PI+); and sperm cells with necrosis: dead cells patented with PI without Annexin V and with complete membrane damage (A-/PI+). The percentages of sperm cells were carefully measured based on the lateral and anterior scattering features.

### Assessment of antioxidant and oxidative biomarkers

After thawing, post-thawed semen samples were centrifuged at 4430 g for 10 min, and then the dilution was split and kept at ˗20 °C. The concentrations of MDA (malondialdehyde), TAC (total antioxidant capacity), and H_2_O_2_ (hydrogen peroxide) were assessed. Likewise, ALT (alanine transaminase) and AST (aspartate transaminase) activities were measured in the extender. All assessments were done using commercial kits (Biodiagnostic, Giza, Egypt) and a spectrophotometer (Spectro UV-Vis Auto, USA) following the instructions provided based on the company.

- Pregnancy rate

A total of 100 multiparous Egyptian buffalo showing spontaneous estrus were randomly designated and inseminated with semen from the GL6, GL6SU, GL3, and GL3SU groups. These extenders were selected based on the post-thawed quality results. Each group (25 female/group) was artificially inseminated with treated frozen/thawed semen. All inseminations were performed by a single AI technician. Pregnancy rates were evaluated using the rectal palpation method on day 60 post artificial insemination.

- Statistical analysis

The data were first statistically examined using two-way analysis of variance (2-way ANOVA; SAS, 2008) to test the main effects of glycerol concentration and sugars (sucrose and trehalose) and their interactions. We found that the effect of glycerol was always significantly dependent on the presence of sucrose or trehalose as factors (Interaction P value < 0.05). Therefore, it was necessary to analyze the data using one-way ANOVA to explore the independent impact of each of the treatments. Post-hoc pairwise comparisons were done using Tukey’s test to correct the P value and account for the multiple hypothesis testing. The pregnancy rates were assessed using the chi-square test (χ2). Differences with *p* < 0.05 were considered significant.

## Results

### Effects of various extenders on sperm quality (%) of equilibrated buffalo bull semen at 5 °C for 4 h

Table 1 shows that there were no statistically significant variations in progressive motility, plasma membrane integrity, and morphological abnormalities of equilibrated buffalo bull semen among all eight extenders (*p* > 0.05). The GL3 group had the highest sperm viability values, followed by GL6SU, while the lowest values were recorded in GL6TR. On the other hand, GL6, GL3SU, and GL3TR groups showed similar results for sperm viability (*p* > 0.05). The sperm viability of the SU and TR groups was comparable, with no significant difference compared to all experimental groups (except the GL3 group).

**Table 1:** Impact of different concentrations of glycerol (GL, 3% or 6%) alone or in combination with either sucrose (SU) or trehalose (TR) on sperm quality (%) of buffalo bull semen after 4 h of equilibration at 5 °C

Treatment^1^	Semen attributes (%)			
	Progressive motility	Viability	Sperm membrane integrity	Abnormality
GL6	75.8 ± 1.54	77.7 ± 1.33^abc^	80.5 ± 1.73	6.3 ± 0.80
GL6SU	78.3 ± 1.67	81.3 ± 1.09^ab^	79.8 ± 1.49	4.7 ± 0.49
GL6TR	72.5 ± 1.12	75.0 ± 1.75^c^	75.5 ± 1.65	5.0 ± 0.86
GL3	79.2 ± 1.54	82.3 ± 1.20^a^	82.3 ± 1.84	4.5 ± 0.34
GL3SU	77.5 ± 2.14	79.7 ± 1.87^abc^	79.8 ± 2.36	6.0 ± 0.82
GL3TR	75.0 ± 1.83	77.2 ± 1.66^abc^	77.7 ± 1.76	6.5 ± 0.85
SU	74.2 ± 1.54	76.0 ± 1.75^bc^	75.3 ± 2.03	5.5 ± 0.89
TR	75.0 ± 2.58	76.0 ± 2.31^bc^	75.5 ± 1.93	5.7 ± 0.76
*P value*	0.17	0.03	0.07	0.45

^1^ Eight Tris extenders supplemented with either 6% glycerol (serve as control group, GL6), 3% (low level, GL3), sucrose (SU, 50 mM), trehalose (TR, 50 mM), 6% glycerol and 50 mM of sucrose (GL6SU) or 50 mM of trehalose (GL6TR), 3% of glycerol and 50 mM of sucrose (GL3SU) or 50 mM of trehalose (GL3TR) were used in this experiment. Within the same column means with different superscripts per treatment (a, b and c) differ significantly (P< 0.05). Level of significance; p< 0.05. Results are presented as mean (%) ±SE, *n *= 6 replicates

### Effects of various extenders on sperm quality (%) of post-thawed buffalo bull semen at 37 °C for 30s

The GL3 group demonstrated the highest values for progressive motility, plasma membrane integrity and viability of buffalo bull semen after thawing (at 37 °C for 30 s; Table 2). While GL3 and GL6SU groups showed the greatest consequences for progressive motility, these improvements were statistically different from the GL6, GL6TR, GL3SU, and GL3TR groups (*p* < 0.05). Both SU and TR groups had the lowest values for progressive motility of buffalo bull semen after thawing (at 37 °C for 30 s; *p* < 0.05). The worst percentages of plasma membrane integrity and viability were reflected in the SU and TR groups. The sperm abnormalities of post-thawed buffalo bull semen (at 37 °C for 30 s) were not affected by the extenders (*p* > 0.05).

**Table 2:** Effect of different concentrations of glycerol (GL, 3% or 6%) alone or in combination with either sucrose (SU) or trehalose (TR) on sperm quality (%) of post-thawed buffalo bull semen at 37 °C for 30s

Treatment^1^	Semen attributes (%)			
	Progressive motility	Viability	Sperm membrane integrity	Abnormality
GL6	38.3 ± 1.67^ab^	41.3 ± 1.54^abc^	39.7 ± 2.03^abcd^	7.3 ± 1.12
GL6SU	44.2 ± 2.39^a^	46.5 ± 2.99^ab^	46.2 ± 2.70^ab^	5.7 ± 0.71
GL6TR	38.3 ± 2.11^ab^	40.3 ± 1.93^bc^	38.3 ± 2.03^bcd^	6.2 ± 0.31
GL3	45.0 ± 1.83^a^	50.0 ± 1.06^a^	48.5 ± 2.54^a^	6.5 ± 0.76
GL3SU	40.0 ± 1.83^ab^	41.8 ± 1.51^abc^	41.8 ± 1.64^abcd^	7.5 ± 0.96
GL3TR	41.7 ± 2.47^ab^	46.0 ± 2.54^ab^	43.7 ± 2.39^abc^	7.8 ± 0.95
SU	34.2 ± 1.54^b^	35.8 ± 1.83^c^	35.8 ± 1.58^cd^	6.8 ± 0.98
TR	34.2 ± 1.54^b^	36.5 ± 1.73^c^	32.8 ± 1.25^d^	7.7 ± 1.31
*P value*	0.001	< 0.0001	< 0.0001	0.67

^1^ Eight Tris extenders supplemented with 6% glycerol (serve as control group, GL6), 3% (low level, GL3), sucrose (SU, 50 mM), trehalose (TR, 50 mM), 6% glycerol and 50 mM of sucrose (GL6SU) or 50 mM of trehalose (GL6TR), 3% of glycerol and 50 mM of sucrose (GL3SU) or 50 mM of trehalose (GL3TR) were used in this experiment. Within the same column means with different superscripts per treatment (a, b, c and d) differ significantly (P< 0.05). Results are presented as mean (%) ±SE, *n *= 6 replicates

### Effects of various extenders on sperm kinematic parameters (%) of post-thawed buffalo bull semen

The lowest values of DAP were observed in the TR groups compared to the other groups (*p* < 0.001), while there was no significant difference compared to the SU and GL6TR groups (Table 3). The GL3 group had the highest values of DCL, while the TR group had the lowest values. The DCL values did not show a significant difference among the GL6, GL6SU, GL3SU, and GL3TR groups (*p* > 0.005). TR had the lowest values of DSL compared to other groups (*p* < 0.001), while there were no significant differences among all the remaining groups (*p* > 0.05). The GL3 group had the highest values of VAP, with no significant difference among all groups (except for the TR group). Further, the VAP in the GL6TR, SU, and TR groups were similar (*p* > 0.05), with the lowest values in the TR group.

The maximum values of VCL were observed in the GL3 group, while the lowest values were noted in the TR group. The TR group had the lowest VSL values compared to the other supplemented groups (*p* < 0.001). However, no significant differences in VSL were detected among all supplemented groups.

The GL3 group had the highest value for VCL, while the TR group had the lowest. The highest values of STR were observed in the GL6TR group, while the lowest values of STR were recorded in the GL3 group. There was no significant difference (*p* > 0.05) among all supplemented groups for STR, except for the GL3 and GL6TR groups. The LIN values were higher in the GL6SU, GL3SU, SU, and TR groups compared to the GL3 group (*p* < 0.05). The lowest values of LIN were detected in GL3 group. No statistical difference was observed in LIN among all groups, except for the GL3 group (*p* < 0.05).

There was a significant decrease in WOB in the GL3 group (*p* < 0.05), while the percentages of WOB did not differ among the other groups (*p* > 0.05). ALH was the highest in the GL3 group compared to other groups (*p* < 0.05; except for the GL6TR and GL3TR groups). No statistical differences for ALH were detected among all groups with the exception for the GL3 group (*p* > 0.05). BCF was significantly lower (*p* < 0.05) in the TR group (except for GL6TR) compared to the other treatment groups. The BCF did not show significant differences among the following groups: GL3SU, GL3TR, SU, GL6, and GL6SU (*p* > 0.05).

**Table 3:** Influence of various concentrations of glycerol (GL, 3% or 6%) alone or in combination with either sucrose (SU) or trehalose (TR) on kinematic parameters of post-thawed buffalo bull sperm at 37 °C for 30s

Group^1^	Semen kinematic parameters^2^										
	DAP (µm)	DCL (µm)	DSL (µm)	VAP (µm/sec)	VCL (µm/sec)	VSL (µm/sec)	STR (%)	LIN (%)	WOB (%)	ALH (µm)	BCF (Hz)
GL6	20.0 ± 1.52^a^	34.2 ± 3.07^ab^	13.3 ± 0.78^a^	46.1 ± 3.39^a^	79.5 ± 7.11^ab^	30.4 ± 1.72^a^	66.0 ± 2.50^ab^	38.5 ± 2.17^ab^	57.8 ± 1.49^ab^	3.1 ± 0.24^b^	22.7 ± 1.49^a^
GL6SU	20.2 ± 0.74^a^	35.6 ± 2.08^ab^	12.2 ± 0.24^a^	47.6 ± 2.66^a^	84.3 ± 6.19^ab^	28.8 ± 0.72^a^	60.8 ± 2.40^ab^	34.3 ± 2.04^ab^	56.8 ± 1.94^ab^	3.5 ± 0.25^b^	22.2 ± 1.46^a^
GL6TR	17.1 ± 1.98^ab^	27.9 ± 3.22^bc^	11.5 ± 0.84^a^	42.4 ± 3.91^ab^	68.0 ± 6.50^bc^	29.0 ± 1.49^a^	69.3 ± 3.41^a^	43.2 ± 3.03^a^	62.5 ± 2.87^a^	3.8 ± 0.26^ab^	19.6 ± 2.49^ab^
GL3	20.8 ± 0.57^a^	40.1 ± 1.89^a^	12.3 ± 0.24^a^	51.5 ± 1.56^a^	97.8 ± 4.96^a^	30.7 ± 0.97^a^	59.2 ± 1.19^b^	31.2 ± 1.68^b^	52.8 ± 2.27^b^	4.6 ± 0.16^a^	22.6 ± 1.52^a^
GL3SU	19.3 ± 0.47^a^	32.5 ± 0.76^ab^	13.3 ± 0.36^a^	45.6 ± 1.31^a^	76.2 ± 2.16^ab^	31.6 ± 1.04^a^	68.8 ± 0.95^ab^	40.8 ± 0.70^a^	59.3 ± 0.56^ab^	3.3 ± 0.12^b^	24.6 ± 0.61^a^
GL3TR	19.2 ± 0.68^a^	32.3 ± 1.64^ab^	12.6 ± 0.62^a^	44.9 ± 1.66^a^	74.6 ± 3.94^ab^	29.7 ± 1.48^a^	65.7 ± 2.09^ab^	39.7 ± 1.80^ab^	60.0 ± 1.29^ab^	3.6 ± 0.22^ab^	22.0 ± 1.49^a^
SU	17.3 ± 0.71^ab^	27.6 ± 1.41^bc^	11.8 ± 0.43^a^	40.8 ± 1.60^ab^	64.5 ± 3.16^bc^	28.0 ± 1.01^a^	68.3 ± 0.84^ab^	43.2 ± 1.14^a^	62.7 ± 1.02^a^	3.0 ± 0.14^b^	20.7 ± 0.92^a^
TR	12.5 ± 0.97^b^	20.8 ± 2.08^c^	8.4 ± 0.60^b^	30.8 ± 3.15^b^	50.5 ± 5.93^c^	20.7 ± 1.96^b^	67.3 ± 2.38^ab^	41.7 ± 2.39^a^	61.2 ± 1.62^a^	3.0 ± 0.43^b^	12.5 ± 1.98^b^
*P value*	< 0.0001	< 0.0001	< 0.0001	0.0001	< 0.0001	< 0.0001	0.01	0.0007	0.006	0.0003	0.0002

^1^ Eight Tris extenders supplemented with 6% glycerol (serve as control group, GL6), 3% (low level, GL3), sucrose (SU, 50 mM), trehalose (TR, 50 mM), 6% glycerol and 50 mM of sucrose (GL6SU) or 50 mM of trehalose (GL6TR), 3% of glycerol and 50 mM of sucrose (GL3SU) or 50 mM of trehalose (GL3TR) were used in this experiment. ^2^ DAP, distance average path (µm); DCL, distance curved line (µm); DSL, distance straight line (µm); VAP, velocity average path (µm/sec); VCL, velocity curved line (µm/sec); VSL, velocity straight line (µm/sec); LIN, linearity (VSL/VCL); STR, straightness (VSL/VAP); WOB, wobble (VAP/VCL); ALH, amplitude of lateral head displacement (µm)and BCF, beat cross frequency (Hz). Within the same column, with different superscripts per treatment (a, b and c) differ significantly (P< 0.05). Results are presented as mean ±SE, *n *= 6 replicates

### Effects of various extenders on sperm quality (%) of post-thawed buffalo bull semen after incubation at 37 °C and 5% CO2 for 2 h

Table 4 illustrates that there were no significant effects of the treatments on the sperm viability (*p* = 0.08), sperm plasma membrane integrity (*p* = 0.06), and abnormalities (*p* = 0.97) after incubation for 2 h at 37 °C and 5% CO_2_. GL3 group had the highest progressive motility of buffalo sperm, while the groups of SU and TR produced the lowest values after incubation at 37 °C and 5% CO_2_ for 2 h (*p* < 0.05). GL6, GL6SU, GL6TR, GL3SU and GL3TR had similar results for progressive motility (*p* > 0.05).

**Table 4:** Effect of various concentrations of glycerol (GL, 3% or 6%) alone or in combination with either sucrose (SU) or trehalose (TR) on sperm characteristics (%) of post-thawed buffalo bull semen after incubation at 37 °C and 5% CO_2_ for 2 h

Treatment^1^	Semen attributes (%)			
	Progressive motility	Viability	Sperm membrane integrity	Abnormality
GL6	34.2 ± 1.54^ab^	37.2 ± 0.95	34.2 ± 1.76	10.2 ± 0.91
GL6SU	38.3 ± 2.79^ab^	40.8 ± 4.15	39.0 ± 4.01	8.8 ± 0.95
GL6TR	32.5 ± 1.71^ab^	35.0 ± 1.71	33.3 ± 2.23	8.7 ± 0.56
GL3	39.2 ± 1.54^a^	41.3 ± 1.54	39.5 ± 2.78	9.0 ± 0.97
GL3SU	35.0 ± 1.29^ab^	38.0 ± 1.46	35.7 ± 0.92	9.8 ± 1.01
GL3TR	35.8 ± 2.71^ab^	38.0 ± 3.79	36.5 ± 3.19	9.5 ± 1.23
SU	30.8 ± 1.54^b^	33.5 ± 1.80	31.7 ± 1.36	9.3 ± 1.20
TR	30.0 ± 1.83^b^	31.5 ± 1.95	29.2 ± 1.56	9.7 ± 1.26
*P value*	0.02	0.08	0.06	0.97

^1^ Eight Tris extenders supplemented with 6% glycerol (serve as control group, GL6), 3% (low level, GL3), sucrose (SU, 50 mM), trehalose (TR, 50 mM), 6% glycerol and 50 mM of sucrose (GL6SU) or 50 mM of trehalose (GL6TR), 3% of glycerol and 50 mM of sucrose (GL3SU) or 50 mM of trehalose (GL3TR) were used in this experiment. Within the same column means with different superscripts per treatment (a and b) differ significantly (P< 0.05). Results are presented as mean (%) ±SE, *n *= 6 replicates

### Effects of various extenders on acrosome reaction of post-thawed buffalo sperm

The impact of different extenders on the sperm acrosome reaction of post-thawed buffalo sperm is clarified in Table 5. The GL3 group had the highest live intact acrosome (LIA, 57%), followed by the GL6SU group (55.3%), and then the GL3SU group (54.7%), without significant differences with the GL6 and GL3TR groups. As expected, the SU or TR group did not protect the LIA from the cryopreservation process. The live detached acrosome (LDA) was the highest in the SU group, followed by the GL3TR group (*p* < 0.05). Dead intact acrosome (DIA) values were greater in the SU and TR groups (*p* < 0.0001) compared to other extenders, while the inferior values were detected in the GL3 and GL6SU groups. There were no significant differences for dead detached acrosome (DDA) among all experimental extenders used in this study (*p* > 0.05).

**Table 5:** Effect of various concentrations of glycerol (GL, 3% or 6%) alone or in combination with either sucrose (SU) or trehalose (TR) on acrosome reaction of post-thawed buffalo bull semen

Treatment^1^	Live sperm with intact acrosome (LIA)	Live sperm with detached acrosome (LDA)	Dead sperm with intact acrosome (DIA)	Dead sperm with detached acrosome (DDA)
GL6	50.0 ± 2.08^ab^	18.3 ± 0.67^ab^	24.7 ± 0.88^cb^	7.0 ± 1.15
GL6SU	55.3 ± 1.20^a^	19.0 ± 0.58^ab^	22.0 ± 2.52^c^	3.7 ± 1.20
GL6TR	44.3 ± 1.45^b^	20.0 ± 1.53^ab^	32.7 ± 2.03^b^	3.0 ± 0.58
GL3	57.0 ± 0.58^a^	17.0 ± 0.58^ab^	22.0 ± 1.15^c^	4.0 ± 1.00
GL3SU	54.7 ± 1.45^a^	16.0 ± 1.53^b^	26.0 ± 1.00^cb^	3.3 ± 0.88
GL3TR	48.7 ± 2.91^ab^	21.7 ± 1.33^ab^	26.7 ± 1.20^cb^	3.0 ± 0.58
SU	31.3 ± 2.03^c^	22.7 ± 0.88^a^	42.3 ± 2.60^a^	3.7 ± 0.33
TR	31.7 ± 3.48^c^	18.3 ± 1.86^ab^	47.0 ± 3.06^a^	3.0 ± 0.58
*P value*	< 0.0001	0.02	< 0.0001	0.06

^1^ Eight Tris extenders supplemented with 6% glycerol (serve as control group, GL6), 3% (low level, GL3), sucrose (SU, 50 mM), trehalose (TR, 50 mM), 6% glycerol and 50 mM of sucrose (GL6SU) or 50 mM of trehalose (GL6TR), 3% of glycerol and 50 mM of sucrose (GL3SU) or 50 mM of trehalose (GL3TR) were used in this experiment. Level of significance; p<0.05. Within the same column means with different superscripts per treatment (a, b and c) differ significantly (P< 0.05). Results are presented as mean (%) ±SE, *n* = 6 replicates

### Effects of various extenders on apoptosis like changes of post-thawed buffalo sperm

The impacts of different levels of GL (3 or 6%) with or without SU or TR on apoptosis-like changes in buffalo bull sperm after thawing are described in Table 6. The highest percentage of viable sperm was recorded in the GL3, GL6, and GL3SU groups compared to the other extenders (*p* < 0.05). The SU and TR groups had the lowest percentages of viable sperm, with no significant difference compared to GL6TR and GL3TR groups (*p* > 0.05). The GL3SU group had the highest percentage of early apoptotic (A+/PI−) sperm (*p* < 0.05), with no significant difference with only the GL3TR group (*p* > 0.05). The percentages of early apoptotic (A+/PI−) were non significantly differ among GL6SU, GL6TR, GL3, and TR groups (*p* > 0.05). The GL3 and GL3SU groups showed significantly lower values (*p* < 0.05) of apoptotic (A+/PI+) sperm compared to the other groups, except for the GL6 and GL6SU groups. The TR group had the highest percentage of apoptotic (A+/PI+) sperm compared to the GL3, GL6, GL6SU and GL3SU groups (*p* < 0.05). The necrotic sperm percentage was superior in the SU group (16.8%), followed by GL6TR (15.5%). GL3SU (11.2%) had the lowest levels of necrotic sperm compared to other groups (*p* < 0.05; except for GL3), followed by GL3 (12.0%).

**Table 6:** Effect of different concentrations of glycerol (GL, 3% or 6%) alone or in combination with either sucrose (SU) or trehalose (TR) on apoptosis-like changes of post-thawed buffalo bull sperm (annexin V/PI assay)

Treatment^1^	Sperm characteristics (%)			
	Viable (A−/PI−)	Early apoptotic (A+/PI−)	Apoptotic (A+/PI+)	Necrotic (A−/PI+)
GL6	42.6 ± 1.44^a^	0.57 ± 0.07^d^	44.6 ± 1.39^cd^	12.2 ± 0.12^ecd^
GL6SU	39.1 ± 0.85^ab^	0.63 ± 0.03^cd^	46.4 ± 0.92^bcd^	13.9 ± 0.18^bcd^
GL6TR	32.3 ± 1.96^bc^	0.87 ± 0.03^bc^	51.3 ± 1.71^abc^	15.5 ± 0.26^ab^
GL3	46.7 ± 0.41^a^	0.83 ± 0.03^bc^	40.5 ± 0.43^d^	12.0 ± 0.03^ed^
GL3SU	46.3 ± 2.54^a^	1.17 ± 0.03^a^	41.3 ± 2.94^d^	11.2 ± 0.61^e^
GL3TR	32.0 ± 1.23^bc^	0.93 ± 0.09^ab^	53.2 ± 1.01^abc^	13.8 ± 0.18^bcd^
SU	28.7 ± 2.20^c^	0.53 ± 0.03^d^	54.0 ± 1.70^ab^	16.8 ± 0.52^a^
TR	29.3 ± 3.09^c^	0.63 ± 0.03^cd^	56.0 ± 3.23^a^	14.1 ± 0.70^bc^
*P value*	< 0.0001	< 0.0001	< 0.0001	< 0.0001

^1^ Eight Tris extenders supplemented with 6% glycerol (serve as control group, GL6), 3% (low level, GL3), sucrose (SU, 50 mM), trehalose (TR, 50 mM), 6% glycerol and 50 mM of sucrose (GL6SU) or 50 mM of trehalose (GL6TR), 3% of glycerol and 50 mM of sucrose (GL3SU) or 50 mM of trehalose (GL3TR) were used in this experiment. Within the same column means with different superscripts per treatment (a, b, c and d) differ significantly (P< 0.05). Results are presented as mean (%) ±SE, *n *= 3 replicates

### Effects of various extenders on TAC, oxidative biomarkers (MDA and H_2_O_2_), and enzymatic activity (AST and ALT) of post-thawed buffalo bull semen

The effects of different levels of GL with SU and TR on TAC, oxidative biomarkers (MDA and H_2_O_2_), and enzymatic levels (AST and ALT) of post-thawed buffalo bull semen are presented in Table 7. The quantities of TAC, AST, and ALT in post-thawed buffalo semen did not demonstrate substantial changes among all treatments (*p* > 0.05). The lowest MDA levels were observed in the SU group compared to the GL6, GL6SU, GL6TR, and GL3 groups (*p* < 0.05). GL6TR (0.082 mM/L) had the lowest level of H_2_O_2_, followed by GL6SU (0.086 mM/L). The highest levels of H_2_O_2_ were noted in the GL3TR (0.128 mM/L) group. Overall, the addition of a low concentration of GL 3% significantly reduced MDA levels, while there was an increase in H_2_O_2_ levels.

**Table 7:** Effect of different concentrations of glycerol (GL) with sucrose (SU) or trehalose (TR) on total antioxidant capacity, oxidative biomarkers, and enzymatic activity of post-thawed buffalo bull semen

Treatment^1^	Oxidative stress markers^2^				
	TAC (mM/L)	H_2_O_2_ (mM/L)	MDA (nmol/mL)	AST (U/mL)	ALT (U/mL)
GL6	0.51 ± 0.03	0.094 ± 0.011^bc^	14.6 ± 1.20^ab^	41.4 ± 1.91	16.0 ± 1.10
GL6SU	0.51 ± 0.02	0.086 ± 0.014^bc^	14.4 ± 0.51^ab^	33.6 ± 2.04	12.8 ± 1.83
GL6TR	0.54 ± 0.04	0.082 ± 0.007^c^	14.9 ± 0.62^a^	37.4 ± 3.01	13.0 ± 0.84
GL3	0.68 ± 0.04	0.128 ± 0.007^ab^	13.6 ± 0.83^abc^	37.0 ± 2.83	13.4 ± 1.03
GL3SU	0.63 ± 0.08	0.124 ± 0.011^abc^	10.6 ± 0.37^cd^	45.6 ± 2.18	12.2 ± 0.97
GL3TR	0.63 ± 0.06	0.138 ± 0.005^a^	11.1 ± 0.90^bcd^	35.0 ± 1.84	13.2 ± 1.32
SU	0.62 ± 0.03	0.092 ± 0.010^bc^	10.0 ± 0.99^d^	41.4 ± 3.88	11.2 ± 0.97
TR	0.65 ± 0.03	0.110 ± 0.005^abc^	11.6 ± 0.47^abcd^	41.4 ± 2.94	11.0 ± 1.34
P value	0.07	0.0004	< 0.0001	0.06	0.16

^1^ Eight Tris extenders supplemented with 6% glycerol (serve as control group, GL6), 3% (low level, GL3), sucrose (SU, 50 mM), trehalose (TR, 50 mM), 6% glycerol and 50 mM of sucrose (GL6SU) or 50 mM of trehalose (GL6TR), 3% of glycerol and 50 mM of sucrose (GL3SU) or 50 mM of trehalose (GL3TR) were used in this experiment. ^2^ Total antioxidant capacity, TAC; Malondialdehyde, MDA; Hydrogen peroxide, H_2_O_2_; aspartate aminotransferase, AST and Alanine transaminase, ALT. Within the same column means with different superscripts per treatment (a, b c and d) differ significantly (P< 0.05). Results are shown as mean ±SE, *n *= 5 replicates

### Fertility trial

**Fig. 1:**
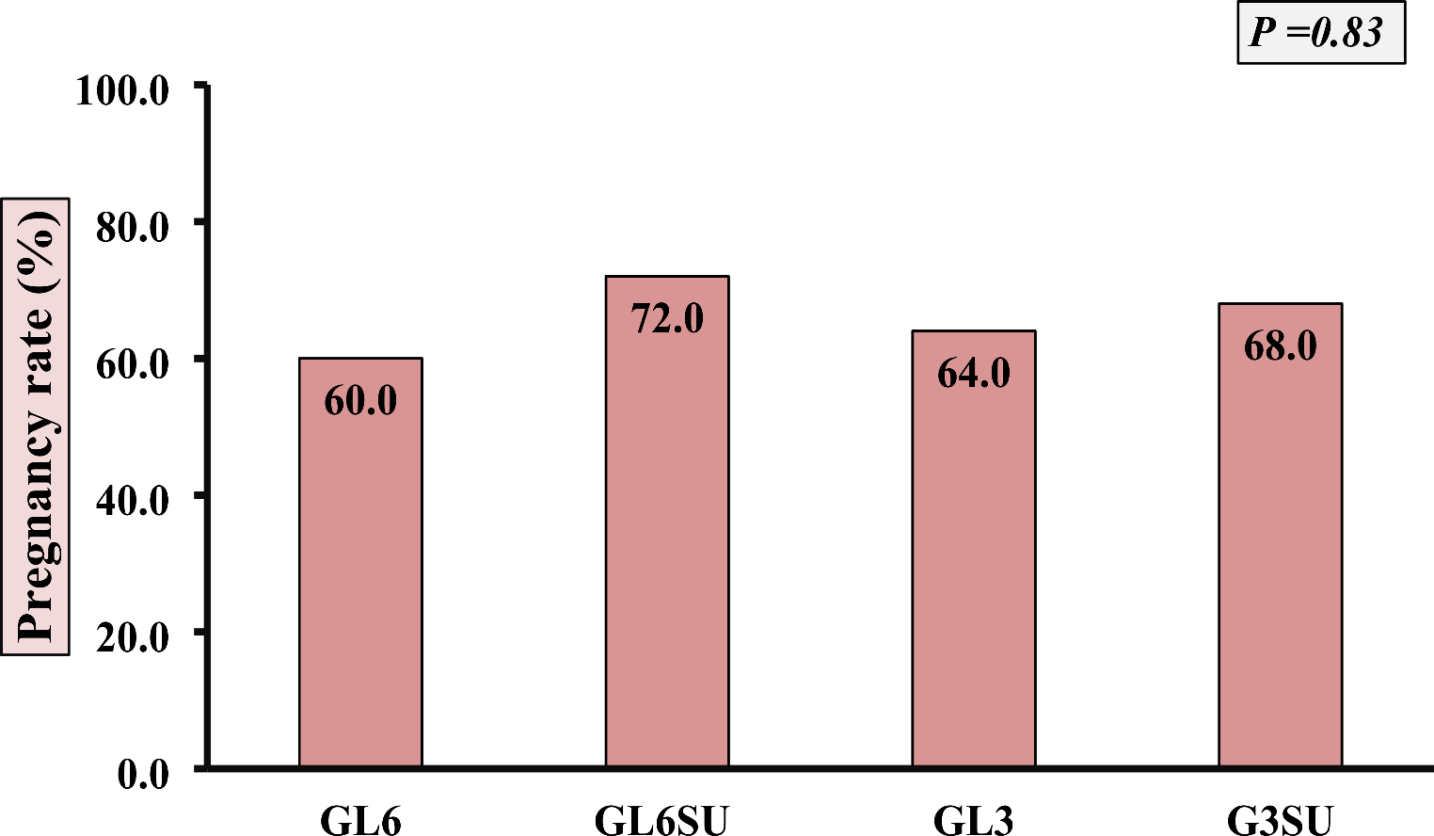
Effect of different concentrations of glycerol (GL) with or without sucrose (SU) on pregnancy rates of Egyptian buffalo. GL6: 6% Glycerol, GL3: 3% Glycerol, SU: 50 mM sucrose

The cryopreserved semen from the GL6, GL3, GL3SU, and GL6SU groups were selected for the fertility trial as they exhibited the best post-thawed sperm parameters. There were no substantial variations observed among the groups in terms of pregnancy rates (*p* = 0.83; Fig. 1). The pregnancy rates for GL6, GL6SU, GL3, and GL3SU were 60%, 72%, 64%, and 68%, respectively. The highest pregnancy rate was noted in the GL6SU group (72%), followed by the GL3SU group (68%).

## Discussion

The current study investigates the comparative effects of low glycerol concentration (3%) and the standard recommended concentration (6%) alone or in combination with non-penetrating cryoprotectants, such as sucrose (SU) or trehalose (TR), in freezing media on the sperm quality, kinematic parameters, apoptosis-like changes, and acrosome status of post-thawed buffalo sperm. The findings suggest that the inclusion of glycerol (3% or 6%) in extenders supplemented with SU or TR can improve post-thawed buffalo sperm quality, including progressive motility, viability, and plasma membrane integrity (except after a 2-hour incubation at 37 °C and 5% CO_2_).

The supplemented extenders did not affect viability, sperm membrane integrity, or abnormality of post-thawed buffalo bull semen after the 2-hour incubation at 37 °C and 5% CO_2_. However, the group with low glycerol addition (GL3) showed better results for sperm progressive motility compared to the extender without glycerol. Kinematic parameters, live sperm with intact acrosome, and viable sperm percentages were significantly improved, while dead sperm with intact acrosome, apoptotic sperms, and necrotic sperms decreased in GL3 group. However, the results for MDA and TAC are not promising and require further clarification. However, further clarification is needed for the results of MDA and TAC. The SU and TR groups showed inferior sperm quality results after thawing. The poor quality of cryopreserved buffalo sperm is a significant concern that hinders the use of artificial insemination in buffaloes (Dalal et al. 2023; Khalil et al. 2023).

Cryobiologists are continually researching the most suitable buffers, energy sources, chemical ideal extenders, and cryoprotectant agents (CPAs) for buffalo sperm preservation. The use of CPAs is essential during semen cryopreservation to minimize the negative effects on sperm health and functionality caused by intracellular ice formation and temperature changes (Mehmood et al. 2017). Determining the optimal doses of CPAs that provide cryoprotective ability is crucial for an effective freezing procedure in water buffalo semen preservation.

Glycerol plays a crucial role in the cryopreservation of buffalo sperm, as studies have shown its ability to improve sperm survival rates during freezing. Overall, glycerol is essential for safeguarding and maintaining buffalo sperm viability during the freezing and thawing process, which is vital for the success of cryopreservation techniques.

Motility is a key parameter for evaluating sperm quality during cryopreservation. Our current research indicates that the extender treatments did not affect the short-term preservation of buffalo bull semen at 5 °C for 4 h. However, after thawing and evaluation at 37 °C for 30 s, the GL3 and GL6SU treatments showed significantly higher progressive motility values compared to the SU and TR groups. The addition of 3% glycerol to the freezing extender resulted in improved viability and membrane integrity in buffaloes compared to the SU and TR groups. Sucrose (SU) has cryoprotective properties and has been shown to effectively freeze human sperm (Hossain and Osuamkpe 2007), minimizing adverse effects. In bulls, Awad (2011) found that using 3% glycerol yielded better results in terms of total and progressive motility. Previous studies have shown that the addition of TR or SU can protect fish sperm plasma membrane, improve cell viability, and reduce MDA levels (Anjos et al. 2021). In contrast to our results, glycerol was found to be toxic to stallion spermatozoa at concentrations equal to or greater than 3.5%, particularly damaging the sperm membranes (Macías García et al. 2012). This may be due to the high sensitivity of spermatozoa from different species.

Computer-assisted sperm analysis (CASA) schemes provide additional evidence regarding velocity categories such as curved line velocity, straight-line velocity, and average path velocity. Consistent with our findings, the addition of 45 mM trehalose and 5% glycerol to the freezing extender improved buffalo sperm kinematics (Iqbal et al. 2018). However, glycerol at low doses (3%) showed a strong affinity with the cells’ phospholipid head clusters during freezing, leading to improved sperm kinematics. A study by Rasul et al. (2007) indicated that a high concentration of glycerol (6%) decreased linear motility and promoted circular motility, suggesting a negative impact of high levels of glycerol on the fine motion properties of buffalo sperm after thawing. These fluctuations may be attributed to glycerol-induced toxic and/or osmotic shocks to buffalo sperm, as observed in ram studies (Rostami et al. 2020). Importantly, there is no existing research in buffalo assessing the combined effect of GL with SU or TR on sperm kinematic variables. Furthermore, the current findings of rapid velocity post-thawing align with plasma membrane integrity, indicating a similar pattern of cryoprotectant effects on the plasma membrane of buffalo spermatozoa after thawing.

The plasma membrane is the primary cause of injury during cryopreservation, leading to decreased viability and fertility. The addition of 3% glycerol (GL) alone or with sucrose (50 mM) resulted in significantly higher percentages of live sperm with intact plasma membranes compared to other groups, with both functionally and structurally intact membranes post-thawing. This trend was also observed in buffalo by Iqbal et al. (2018). Additionally, the percentage of sperm with detached acrosomes was lower in all groups supplemented with 3% or 6% GL, while the non-supplemented group had a higher percentage of detached acrosomes. The protective effects of cryoprotectants, particularly sucrose or trehalose, are attributed to their ability to stabilize the plasma membrane by interacting with the polar heads of membrane phospholipids (Anchordoguy et al. 1987), thereby modulating membrane fluidity (Swelum et al. 2011).

These results are consistent with previous studies on buffalo bulls (Badr et al. 2010; Iqbal et al. 2018; Rasul et al. 2007). In contrast, a study by Bakhsh et al. (2023) found that 7% GL yielded better results for sperm motility and kinematics in bulls compared to 5% or 6% GL. Sucrose also helps control cell dehydration and the crystallization pattern of solute channels in unfrozen water, reducing ice crystal formation. GL may interact with membrane phospholipids to create a hypertonic environment (Anchordoguy et al. 1987), minimizing plasmalemma injury during the freeze-thaw process (Blazek et al. 2015). The stability of the sperm membrane may explain the positive impact of low GL levels in maintaining sperm membrane and acrosome integrity, reducing apoptotic sperm, and consequently enhancing sperm fertilizing capability, as demonstrated in our study.

The regular acrosome membrane is a crucial indicator of sperm acrosome reaction before fertilization (Tello-Mora et al. 2018). The cryopreservation process triggers cryo-capacitation, leading to the destabilization of the sperm plasma membrane through calcium influx and phosphorylation of tyrosine proteins. This can result in acrosome detachment, leading to sperm dysfunction. In this study, GL (3% or 6%) alone or in combination with SU and TR improved the acrosome membrane integrity after the post-thawing process. The addition of trehalose, along with other cryoprotectants in a Tris–citric acid-based extender, resulted in a higher percentage of spermatozoa with intact acrosomes. Previous studies have shown that GL alone or in combination with SU/TR reduced the susceptibility of cryopreserved spermatozoa to undergo acrosome reaction in the presence of lysophosphatidylcholine, a phospholipid that is more prevalent in buffalo spermatozoa (Iqbal et al. 2016). In another experiment, Thananurak et al. (2019) found that the supplementation of SU had more beneficial effects than raffinose in cryopreserved chicken sperm, with a fertility rate of 91.16% compared to 70.38%.

Apoptotic sperm death involves biochemical and structural changes in dying sperm (Khalil et al. 2024). Cryopreservation can increase apoptosis-like changes in buffalo sperm (Abdelnour et al. 2023; Khalil et al. 2024). Cryo-stress reduced mitochondrial function and induces apoptosis in buffalo sperm (Dalal et al. 2023). Research suggests that reducing calcium and cholesterol effluxes or inhibiting tyrosine phosphorylation can mitigate capacitation-like changes during cryopreservation (Dalal et al. 2023). Our findings indicate that GL (3%) alone or with SU (50mM) boosted sperm viability and reduced apoptotic sperm compared to SU and TR groups, suggesting GL’s potential to protect cryopreserved sperm by modulating calcium and cholesterol effluxes. The present results indicate that the in vivo pregnancy rates were higher in GL6SU (72%) followed by GL3SU (68%) indicating that higher doses of GL had greater results in the in vivo fertility trial. Further research in this area is still necessary to determine a better combination of cryoprotectants to enhance the in vivo fertility based on the in vitro trial. Improving the fertility of buffalo sperm is crucial for enhancing the reproductive and productive competence of water buffalo. The experiment by Iqbal et al. (2018) significantly found improvement in the in vivo fertility of buffalo cows inseminated with semen cryopreserved with TR (45mM) and GL (3%).

## Conclusions

This study demonstrates that the addition of low concentrations of glycerol (3%), either alone or in combination with sucrose or trehalose, to the freezing extender does not have a negative impact on post-thaw buffalo sperm characteristics. The Tris-based extender enriched with 3% glycerol showed higher values for progressive motility, sperm kinematics, sperm membrane integrity, and viability of post-thawed semen (37 °C for 30 s) and reduced MDA levels compared to the extender with 6% glycerol. This suggests the possibility of reducing glycerol (3%) levels in freezing extenders for buffalo sperm. However, the specific beneficial effects of low glycerol concentrations in freezing extenders for buffalo sperm require further investigation, particularly through molecular studies.

## Data Availability

All data generated or analyzed during this study are included in this published article.
